# Does distrust in providers affect health-care utilization in China?

**DOI:** 10.1093/heapol/czw024

**Published:** 2016-04-26

**Authors:** Jane Duckett, Kate Hunt, Neil Munro, Matt Sutton

**Affiliations:** ^1^School of Social and Political Sciences, University of Glasgow, Glasgow G12 8RT, UK; ^2^MRC/CSO Social and Public Health Sciences Unit, University of Glasgow, Glasgow G2 3QB, UK; ^3^Manchester Centre for Health Economics, Institute of Population Health, University of Manchester, Manchester M13 9PL, UK

**Keywords:** Trust, utilization of health services, clinics, China

## Abstract

How trust affects health-care utilization is not well-understood, especially in low- and middle-income countries. This article focuses on China, a middle-income country where low trust in health-care settings has become a prominent issue, but actual levels of distrust and their implications for utilization are unknown. We conducted a nationally representative survey of the Chinese population (November 2012 to January 2013), which resulted in a sample of 3680 adult men and women. Respondents rated their trust in different types of health-care providers. Using multivariate logistic and negative binomial regression models, we estimated the association between distrust in clinics and respondents’ hospital visits in the last year; whether they had sought hospital treatment first for two common symptoms (headache, cold) in the last 2 months; and whether they said they would go first to a hospital if they had a minor or major illness. We analysed these associations before and after adjusting for performance evaluations of clinics and hospitals, controlling for sex, age, education, income, insurance status, household registration and self-assessed health. We found that distrust in hospitals is low, but distrust in clinics is high and strongly associated with increased hospital utilization, especially for minor symptoms and illnesses. Further research is needed to understand the reasons for distrust in clinics because its effects are not fully accounted for by poor evaluations of their competence.

Key Messages

- There has not been a general collapse of public trust in health-care providers in China, but distrust in clinics is high.
- Distrust in clinics is strongly associated with greater hospital utilization, even for minor symptoms such as colds and headaches.
- Distrust in clinics is not simply due to perceptions that they are less competent.
- The strong and consistent associations between distrust and utilization in our study indicate the need for research in other middle- and low-income countries.

## Introduction

The importance of trust in health-care settings in North America and Western Europe is now well-established (Mechanic 1998; Hall *et al*. 2001; Gilson 2003), and research has begun to extend to low- and middle-income countries (Gilson 2005; Molyneux *et al*. 2005; Riewpaiboon *et al*. 2005; Schneider 2005; Lee 2011; Ozawa and Walker 2011; Tam 2012). The effects of trust on utilization, however, are still not well-understood (LaVeist *et al*. 2009). Research in the USA suggests that distrust in health-care settings decreases utilization or affects choice of providers (LaVeist *et al*. 2000 , 2009; O'Malley *et al*. 2004; Ostertag *et al*. 2006), but the relationship between trust, distrust and utilization has not been the subject of focussed research in the developing world.

Our article analyzes data on distrust and utilization from a nationally representative survey conducted in China, a middle-income country where distrust is seen as a major problem for the health-care system (Lee 2011). Although leading international researchers have argued there has been a collapse of public trust in China (Hsiao and Hu 2011; Lee 2011), the extent of public distrust has not been empirically established. There has been research on ‘patient’ distrust in physicians—seen as the cause of rising medical disputes, stress in medical professionals (Huang *et al*. 2012) and physical attacks on doctors (Gong and Zhang 2006; Zheng 2007). These patient studies, however, can only report on people who have actually sought diagnosis or treatment (Yang and Yang 2009; Tam 2012); they cannot determine levels of distrust in the general population and how it affects their health-care seeking behaviour.

Qualitative research in China indicates that distrust of health-care providers (by which we mean facilities, such as clinics and hospitals, that deliver health services) may deter people from seeking care (Zhang *et al*. 2007; Lora-Wainwright 2011), and some have argued—but with scant evidence—that distrust in clinics leads to low utilization of their services or inappropriately high use of hospitals (Yang and Yang 2009; Bhattacharyya *et al*. 2011; Hu *et al*. 2011; Liu *et al*. 2011; Wang *et al*. 2011; Yip *et al*. 2012). Yet to date, studies of health-care utilization in China have focussed on socio-demographic factors, income, health insurance protection and distance from health service providers (Wagstaff *et al*. 2009).

Our study is the first to use population survey data to examine distrust in clinics and public hospitals and its relationship with health-care utilization. Its findings will be of interest to researchers and policy makers in China and those with an interest in health-care in other middle- or low-income countries. As we discuss below, sources of distrust in China seem to differ significantly from those in North America and Western Europe, and they are increasingly likely to be the found elsewhere as China’s model of development influences low-income nations (Zhao 2010). Its influence on health systems in Africa, for example, is likely to grow following recent agreements to deepen health cooperation (FOCAC 2012).

### A focus on distrust in types of health-care providers

We conceptualize distrust as either a low level or lack of trust, with trust in turn defined as the expectation that a person or institution will act in one’s best interests (Hall *et al*. 2001). Rather than examining ‘personal’ distrust of individual health-care professionals, we examine ‘institutional’ distrust (Mechanic 1996; Gilson 2003). Capturing institutional distrust is appropriate given our study’s interest in the general population, including those who have not sought diagnosis or treatment as well as those who have. Institutional distrust can include a generalized abstract distrust in institutions as well as ‘concrete perceptions about institutions with which patients have had experience’ (Mechanic 1996). Generalized abstract distrust may be shaped by many factors, such as the experiences of family, friends and neighbours, or media reports, especially for respondents who have not had direct experience. In addition to these factors, people with personal experience may add elements of trust or distrust in individual doctors or particular institutions.

We focus on different types of health-care providers as a particular sub-set of health-care institutions, because it is at this (provider) level that distrust is thought to influence utilization in China (Yip *et al*. 2012). Chinese people seeking diagnosis and treatment have a wide range of provider choices, especially if they are paying out of pocket [and individual direct payments remain high at 34% of total health expenditures in 2012 (Ministry of Health 2013)]. They may often base their choice not on personal trust in individual doctors, but on their perceptions, and trust or distrust, of particular types of provider. Although some people in China try to identify trustworthy doctors through personal connections, this option is likely to be available only to those with high levels of social capital (Lora-Wainwright 2011).

### Study context: China’s health reforms before and since 2009

China’s health-care ‘reforms’ are considered to have begun in the 1980s, when central government policies enabled public health-care providers to generate income from certain procedures and medicines, permitted private practice, and by the turn of the 21st century allowed the privatization of some hospitals. Although most hospitals and many primary care providers remained under public ownership, their share of income from charging fees increased and their share of income from government budgets decreased to as little as 10% (Duckett 2011). The many problems that this commercialization of provision caused have been researched and reported by others, and they include factors that are thought to have led to growing distrust in China’s health service providers: perceptions of doctors and providers as motivated to generate income—especially from fees for unnecessary diagnostic tests and expensive medicine prescriptions—rather than to do what is best for patients; low public investment in clinics and the lower tiers of the health system; and poorly trained primary care physicians (Liu 2004; Blumenthal and Hsiao 2005). While research has linked providers’ income generation incentives to a perceived general collapse in public trust, for clinics, community and town or township facilities, poor training and facilities are also thought to have played a role, discouraging their use and leading to unnecessary hospital utilization (Hsiao and Hu 2011; Yip *et al*. 2012).

In April 2009, however, the Chinese central government published an ambitious new programme aimed at tackling the many problems in the health-care system (Party Central Committee and State Council 2009). It included measures to solve problems often cited as causing distrust in providers: their reliance on fee-charging and medicine sales for income, and low quality primary care. Primary care providers were, for example, to be prevented from adding mark-ups to drugs they sell, and in its first 3-year phase, the programme was to target substantial amounts of funding at primary health-care, improve their staff training and tie funding to performance (Yip *et al*. 2012).

The effects of the 2009 programme are still not well-understood. An early external review of its first 3 years found impressive progress in some areas. But it also reported that the limited data on implementing primary care and public hospital reform measures indicated no change in patients over-utilizing hospitals and making less use of primary care facilities (Yip *et al*. 2012). Our survey, conducted between 1 November 2012 and 17 January 2013, therefore provides important nationwide data on patterns of health-care utilization just over 3 years after the 2009 programme began, as well as new data on distrust that allows us to examine its associations with utilization.

## Methods

### Nationally representative survey

We use data from a nationally representative survey we designed and commissioned. We obtained ethical approval through the University of Glasgow, and we also obtained the free and informed consent of the subjects. Our survey questionnaire asked about respondents’: (i) life circumstances; (ii) health and health-care utilization; (iii) health-care performance evaluations, values and trust and (iv) income and expenditure. The survey was conducted by the Research Centre for Contemporary China (RCCC) at Peking University, with whom we worked closely. Its target population was mainland Chinese citizens aged 18–70 years residing for >30 days in family dwellings in all 31 provinces. The survey used the GPS ‘assisted area sampling method’ to project a grid onto 2855 counties, county-level cities or urban districts of the same status (Landry and Shen 2005). This method has been pioneered by RCCC, and is internationally recognized as enabling robust representation of China’s large population (see Supplementary Appendix S1). Post-fieldwork stratification and weighting by age and gender was based on population statistics from the 2010 census (Table 1). The result was a sample of 5424 dwellings in which 3680 valid interviews were completed, giving a response rate of 67.8%. This is similar to the response rates of other randomly sampled public surveys in China. (For information on questionnaire design, pilot and quality controls, see Supplementary Appendix S2.)

**Table 1.** Sample characteristics (age, sex) compared with 2010 census

	2010 census^a^		Unweighted sample		Weighted sample	
	Male	Female	Male	Female	Male	Female
Age	%	%	%	%	%	%
18–29	27.3	27.7	18.6	17.3	27.3	27.7
30–39	22.0	21.8	18.4	19.6	22.0	21.7
40–49	23.5	23.4	24.0	24.5	23.5	23.4
50–59	16.3	16.3	18.4	19.0	16.3	16.3
60–70	10.9	10.9	20.7	19.5	10.9	10.9

Source: China National Health Attitudes Survey 2012–13 (Fieldwork 1 November 2012—17 January 2013. *N* = 3680), and Census Office of the State Council *et al.* (2012, p. 4, Table 3-1a).

### Independent variable: distrust

Studies of trust and distrust in health-care settings have used a wide range of measures, and have developed different scales to gauge trust and distrust in doctors, health-care providers, the medical profession, health insurers and health-care systems as a whole (Hall *et al*. 2001; Goudge and Gilson 2005). These previous studies and measures, however, have been developed mainly in North America and Western Europe, and do not transfer well to China. Many, for example, focus on physicians and use the ‘Trust in Physicians Scale’ (TIPS). But the TIPS assesses patients’ level of trust in their individual physician, and therefore does not work well where people do not interact with the health-care system primarily through one individual physician (LaVeist *et al*. 2009). Such is the case in China, where people normally do not register with a particular doctor, but instead may attend a facility of their own choosing. Although their insurance may be tied to a particular provider, it often requires high co-payments and so patients may choose to go elsewhere and pay on a fee-for-service basis.

In any case, multi-item scales developed in the USA to measure distrust in health-care systems lack cross-cultural validity and so do not work well in the Chinese context. LaVeist *et al*. (2000), for example, used a five item scale to measure ‘mistrust in hospitals’ that included questions—about privacy and medical experiments—that are not salient in China, but excluded factors, such as financial incentives for doctors or poor skills of some physicians, that are thought to have a strong effect (LaVeist *et al*. 2000).

Given the problems using multidimensional scales and the fact that trust has been under-researched in China, we chose to use a single, holistic measure encapsulated in a direct question. Here, we drew on Hall *et al*.s’ (2001) conclusion—based on an extensive review of the literature—that trust in health-care contexts has a ‘distinctly holistic’ quality. Adjusting for our focus on types of health-care providers, we asked: ‘To what extent do you trust the following institutions to look after your health-care needs?’ We translated ‘trust’ into Chinese as ‘*xinren*’, the term overwhelmingly used by researchers, the media in China, and participants in our focus group discussions to discuss trust in health-care contexts (Hu *et al*. 2011; Huang *et al*. 2012). Based on our conceptualization of distrust as either a low level or lack of trust, we created a scale using the response categories: ‘trust a lot’, ‘trust somewhat’, ‘don’t trust much’, ‘don’t trust at all’ for different types of health-care providers.

Our interviewers showed survey respondents a list of the main types of health-care providers available in China: small clinics; village health clinics; ‘community health service stations;’ township, town or neighbourhood health centres; county/city/district hospitals; city/prefecture hospitals; province level hospitals; and pharmacies. The number of facilities a respondent scored varied depending on the providers locally available. Through factor analysis, we found that trust (and distrust) in different types of facilities clustered to produce a two-factor solution, with clinics, community, township and town health service providers correlating highly on the one hand (Cronbach’s α 0.89), and different types of hospital on the other (Cronbach’s α 0.90) (Supplementary Table S1). We computed two summary distrust scores: one for the four types of highly correlated community providers, hereafter referred to as ‘clinics’, and one for the three types of hospital. To do this, we recoded the responses to our trust question so that one indicated least distrust and four most distrust—and then averaged them across ‘clinics’ and, separately, ‘hospitals’. We imputed scores only if the entire summary measure was missing (4.7% or 173 of 3680 respondents for clinics; 7.3% or 268 of 3680 respondents for hospitals (see Supplementary Appendix S3 in the supplementary material for more information on how we handled missing data).

To check the validity of responses to our trust questions, we tested whether they correlated with perceptions of unethical practices as well as with performance evaluation measures. In our survey, we had asked about the perceived likelihood of a range of ‘unethical’ practices: ‘prescribing medicine not covered by insurance even when alternatives covered by insurance are available’; ‘requiring comprehensive tests even when the diagnosis is perfectly clear’ and ‘taking informal payments for treatment which has already been paid for’. We constructed an index of unethical practices by averaging the likelihood of the three types of practices. Our measures of distrust in clinics and hospitals correlate with perceived likelihood of unethical practices at 0.19 and 0.16 respectively (both *P* < 0.001). (We used the Pearson correlation coefficient consistently throughout this study to facilitate comparisons between correlations). Correlations of the distrust measures with performance evaluations of clinics and hospitals are negative, as we would expect. Distrust in clinics correlates negatively with evaluations of clinics’ value for money at −0.39, and with clinic competence at −0.45 (both *P* < 0 .001). Distrust in hospitals correlates negatively with hospital value for money at −0.24, and with hospital competence at −0.38 (both *P* < 0.001).

### Dependent variables

We enquired about several dimensions of health-care utilization. We asked two questions about people’s actual utilization. First, we asked all respondents how many times they had visited a hospital for their own health in the last year. Second, we measured recent behaviour for two common, usually self-limiting, symptoms: cold and headache. We asked: ‘In the last 2 months, have you suffered from [cold, headache]?’ and if so, ‘Did you go to see anyone for advice or medicines for it?’ If yes, respondents indicated where they went first, selecting from a list of facilities. These measures for choice of provider are available only for respondents who experienced each of these symptoms over the index period. To capture intentions, we asked all respondents ‘Where would you go first for diagnosis and treatment if you thought you had

1. … a minor illness, one which might require a short course of treatment?
2. … a major illness, one which might require an extended course of treatment?’

The wording of the question in Chinese made clear that we were asking what the respondent’s first choice would be of a facility to visit first (in time). In answering questions about actual and intended health-care utilization, respondents chose from the same list of health service providers as used in the questions about trust. In addition, respondents could choose an ‘other’ type of facility, and for the questions about intended behaviour they could choose ‘a medical institution nearby, but not sure which level it is’.

Our rationale for including questions about intended as well as actual utilization was to investigate the potential behaviour of people who had not had recent experience of the health-care system. We asked about ‘minor’ symptoms and illnesses because they would not normally need a hospital visit and so are a more robust test of the association between distrust in clinics and hospital utilization. They also enable us to identify possibly ‘unnecessary’ hospital utilization.

### Analysis

We adjusted for standard socio-economic or ‘predisposing’ characteristics (Andersen 1968; 1995): sex, age, education level and self-assessed health status. We measured age in five deciles, with 18–29 as the youngest group, and over 60 as the oldest. We did not measure age in years because Chinese survey respondents tend to answer questions about age by referring to the most recent 10-year milestone they have passed. Thus, ‘I’m forty’ often means ‘I’m past forty’, producing spikes in the age distribution at each decile rather than data on actual age distribution. We measured education in nine categories, which we collapsed into a four point scale consisting of: primary school or less, middle school, senior high school and/or technical college, and university (undergraduate to PhD). We asked respondents to assess their physical health over the last 12 months using a four point scale that we recoded so that higher values equate to better self-assessed health.

We also controlled for respondents’ ‘enabling’ characteristics: income, household registration and insurance. We present self-reported household income as a continuous variable that we equivalized using a modified OECD equivalence scale, converting the units to thousands of yuan (Forster 2004). Another enabler is ‘non-agricultural registration’—something that strongly influences people’s life chances, as well as the types of insurance they can have (such as urban employee and urban residents’ insurance, instead of the usually much less generous rural cooperative medical schemes) (Wang 2005a). We created indicators for having ‘non-agricultural’ (as opposed to ‘agricultural’) registration and insurance.

We included a covariate for ‘convenience’—a commonly expected influence on utilization. Utilization studies sometimes use the distance from a respondent’s home or workplace to any health service provider as a proxy for convenience of access. However, the obstacles presented by distance can vary depending on the modes of transport available, the respondent’s ability to use them (e.g. being fit enough to cycle), traffic congestion and other conditions, so we adopted respondents’ own evaluations of providers’ ‘convenience’ as a direct subjective measure. We then added two further sets of performance evaluation controls: perceptions of providers’ value for money; and the skills and experience of their staff, which we refer to as ‘competence’, each coded from one (‘very bad’), to four (‘very good’). We conducted a factor analysis to confirm that evaluations of convenience, value for money and competence clustered for the two groups of providers (‘clinics’ and ‘hospitals’) (see Supplementary Table S2 for factor loadings and Cronbach’s α scores). We then constructed a composite score for each dimension of performance evaluation by averaging the items with factor loadings above 0.60.

We controlled for the clustered structure of our data and possible local differences in health services provision by including in our analysis a random effect at county level (Raudenbush and Bryk 2002). This allows for the fact that our 60 primary sampling units are not the complete population of all China’s counties and districts, of which there are more than 2000, but rather a random sample. It also allows for local differences in provision that may, in China’s decentralized fiscal system, result from variation in local (government, insurance or out-of-pocket) health spending (Wong 2009), the nature of rural cooperative insurance schemes that are organized at county level, or local policy experimentation, also often organized by county.

We also considered the possibility that distrust in health-care providers could be influenced by political fear or could reflect general patterns of institutional distrust in China. Although there are continuing debates in political science and sociology about whether or not there are culturally or politically induced response biases in China, most scholars who have looked at this issue in detail reject the notion that expressed political trust is merely a reflection of political fear (Shi 2001; Steinhardt 2012). In support of this, studies have shown that while Chinese citizens tend to report high trust in central political authorities, their trust in local government and in the news media is lower (Li 2004; Wang 2005b). We asked our respondents a standard battery of institutional trust questions and included in our analysis three items—distrust in central government and the Chinese Communist Party (CCP) (these are combined because they correlate at 0.87), distrust in local government and distrust in the news media.

Whether or not people distrust can be due in part to personality traits or inclination (Mechanic 1996; Goudge and Gilson 2005), and some research has linked declining trust in medical institutions to declining general trust across populations, measured using a question about whether ‘most people can be trusted’ (Mechanic 1996). We therefore included in our model a control for ‘general distrust’ using a widely used public opinion survey instrument: ‘Generally, do you think people can be trusted or do you think that you can't be too careful in dealing with people?’ (Bjornskov 2007; Steinhardt 2012).

We first conducted exploratory analysis (using Pearson correlations) to determine the levels of distrust across the population in different providers. We found distrust in hospitals to be low, but distrust in ‘clinics’ (which includes village, community, town and township providers) to be high and significantly correlated with both low clinic utilization and high hospital utilization. Because overutilization of hospitals is considered a serious problem in China’s health-care system, we then focussed on the associations between distrust in clinics and hospital utilization. We tested a series of models relating distrust to health-care utilization, using logistic regression for all of the dependent variables except for number of hospital visits, for which we used negative binomial regression. First, we analysed the associations between our composite measure of distrust in ‘clinics’, respondents’ personal (predisposing and enabling) characteristics, and utilization of hospitals (Model 1; results presented in Table 4). Then we added evaluations of clinics to establish whether the effects of distrust in clinics are robust once we take into account respondents’ views on their competence, convenience and value for money (Model 2; results presented in Table 5). Next, we added the same evaluations for hospitals to test whether they could better account for decisions about hospital use (Model 3, Table 5). Finally, we added measures of distrust in government and the Party, in the media and in people in general, to test whether the effects of distrust in clinics are due to its correlation with respondents’ distrust in institutions or in people in general (Model 4, Table 5). We also ran analyses separately for those who had said that their minor condition had required no change in activities as a further test of health seeking behaviour relating to less serious conditions. Because social health insurance, available to the mostly urban residents who have ‘non-agricultural’ household registration, provides more generous cover than the cooperative medical schemes available to the mostly rural residents with ‘agricultural’ registration, we ran the analyses separately for those with agricultural and non-agricultural registration (results reported in Supplementary Tables S4 and S5).

**Table 2.** Trust and distrust in types of health-care providers

	Trust a lot	Trust somewhat	Don't trust much	Don't trust at all	Don't know	No answer
	%	%	%	%	%	%
Small clinic (*zhensuo*)	6.6	55.3	25.8	2.3	8.4	1.7
Village health clinic	5.4	51.7	21	2.2	7.5	2.2
Community health service station	4.5	50.6	16.1	0.9	25.7	2.1
Township, town or street health centre	6.1	54.8	14.5	0.9	21.2	2.6
County/city/district hospital	19	62.4	6.7	0.5	9.5	1.9
City/prefecture hospital	25.6	53.8	3.9	0.3	14.6	1.9
Province level hospital	30	46.4	3.6	0.3	17.7	2.1

Source: as in Table 1.

**Table 3.** Health-care behaviour, predisposing and enabling characteristics, and attitudes

	*N*	%	Mean^a^	SD	Range	% dk/ na^b^
*Actual health-care behaviour*						
*N* hospital visits in last year	3680		0.55	1.33	0–24	0
Went to hospital for cold symptoms	1267	7.6	na	na	0 no, 1 yes	0^c^
*N* days changed daily activities due to cold symptoms	1246		1.48	3.76	0–31	0.6^c^
Went to hospital for cold but no change of activities	893	6.5	na	na	0 no, 1 yes	0^d^
Went to hospital for headache	700	9.4	na	na	0 no, 1 yes	0^c^
N days changed daily activities because of headache	682		1.84	4.77	0–31	0.5^c^
Went hospital for headache but no change of activities	480	7.5	na	na	0 no, 1 yes	0^d^
*Intended health-care behaviour*						
Would go to hospital for minor illness	3680	13	na	na	0 no, 1 yes	0
Would go to hospital for major illness	3680	88	na	na	0 no, 1yes	0
*Predisposing and enabling characteristics*						
Male	3680	50	na	na	0 no, 1 yes	0
Age in deciles	3680		3.61	1.33	2 (18–29)–6 (60+)	0
Education	3680		2.17	0.97	1 primary–4 university	0
Self-assessed health	3670		2.81	0.9	1 very poor–4 very good	0.3
Equivalized annual household income, thousand yuan	2772		23.5	24	2–200	24.7
Holds any type of health insurance	3680	92	na	na	0 no, 1 yes	0
Non-agricultural household registration	3665	37	na	na	0 no, 1 yes	0.4
*Distrust and performance evaluations*						
*Clinics*						
Distrust clinics	3507		2.19	0.51	1 trust a lot–4 not at all	4.7
Value for money of clinics	3039		2.84	0.58	1 very bad–4 very good	17.4
Convenience of clinics	3282		3.43	0.57	1 very bad–4 very good	10.8
Competence of clinics	3253		2.74	0.55	1 very bad–4 very good	11.6
*Hospitals*						
Distrust hospitals	3412		1.78	0.51	1 trust a lot–4 not at all	7.3
Value for money of hospitals	2870		2.96	0.67	1 very bad–4 very good	22
Convenience of hospitals	3083		2.46	0.76	1 very bad–4 very good	16.2
Competence of hospitals	3084		3.36	0.49	1 very bad–4 very good	16.2
*Levels of distrust in non-health-care institutions*						
Distrust central government/CCP (average)	3389		1.64	0.6	1 trust a lot–4 not at all	7.9
Distrust local government	3289		2.35	0.8	1 trust a lot–4 not at all	10.6
Distrust media	3343		2.24	0.66	1 trust a lot–4 not at all	9.2
Distrust people in general	3680	40	na	na	0 no, 1 yes	0

Source: authors’ survey as reported in Table 1.

^a^For categorical variables, means and standard deviations are not applicable; all other variables are treated as covariates.

^b^Don’t know, no answer.

^c^Per cent of those reporting symptoms.

^d^Per cent of those reporting symptoms that did not cause them to change their daily activities.

**Table 4.** Relationships of personal characteristics and distrust in clinics to hospital utilization (Model 1)

	N hospital visits		Went to hospital for cold		Went to hospital for headache		Would go to hospital for minor illness		Would go to hospital for major illness	
	Event rate ratio			Odds ratios with 95% CI (lower, upper)						
Male	1.04	(0.92,1.18)	1.00	(0.70,1.44)	1.16	(0.73,1.85)	0.94	(0.79,1.14)	0.92	(0.78,1.08)
Insured	1.48	(1.02,2.154	1.71	(0.68,4.32)	1.60	(0.63,4.08)	1.64	(1.08,2.49)	1.50	(1.02,2.21)
Non-agricultural h/h reg	1.42	(1.09,1.85)	1.98	(1.25,3.14)	1.18	(0.65,2.17)	2.11	(1.56,2.87)	1.65	(1.15,2.37)
Age in deciles	1.10	(1.03,1.18)	1.05	(0.91,1.23)	1.01	(0.85,1.20)	1.01	(0.93,1.10)	0.94	(0.86,1.02)
Self-assessed health	0.60	(0.53,0.67)	0.77	(0.60,1.01)	0.82	(0.64,1.07)	1.02	(0.90,1.15)	0.97	(0.86,1.09)
Education	1.11	(1.02,1.21)	1.09	(0.78,1.53)	1.06	(0.80,1.40)	1.16	(0.99,1.36)	1.09	(0.92,1.30)
Household income	1.00	(1.00,1.01)	1.01	(1.00,1.02)	1.01	(1.00,1.02)	1.00	(1.00,1.01)	1.00	(0.99,1.01)
Distrust clinics	1.19	(1.01,1.40)	1.81	(1.10,2.98)	2.42	(1.35,4.32)	1.90	(1.43,2.52)	1.53	(1.14,2.04)
Intercept variance										
For null model	0.40		0.65		0.70		0.91		0.38	
For fitted model	0.34		0.43		0.61		0.66		0.37	

Source: authors’ survey as reported in Table 1.

**Table 5.** Testing robustness of the relationship of distrust in clinics to hospital utilization

	*N* hospital visits		Went to hospital for cold		Went to hospital for headache		Would go to hospital for minor illness		Would go to hospital for major illness	
	Event rate ratio			Odds ratios with 95% CI (lower, upper)^a^						
*Model 2*										
Distrust in clinics	1.18	(1.00,1.39)	1.60	(0.85,3.05)	2.30	(1.13,4.69)	1.86	(1.40,2.47)	1.56	(1.18,2.06)
Clinic VFM^b^	1.05	(0.86,1.28)	1.17	(0.66,2.07)	1.33	(0.78,2.26)	1.38	(1.13,1.69)	1.08	(0.81,1.43)
Clinic convenience	0.95	(0.81,1.11)	0.66	(0.41,1.06)	1.12	(0.74,1.70)	0.78	(0.65,0.94)	1.10	(0.90,1.35)
Clinic competence	0.95	(0.78,1.16)	0.66	(0.38,1.15)	0.64	(0.36,1.14)	0.73	(0.55,0.98)	0.95	(0.71,1.28)
Intercept variance	0.33		0.40		0.58		0.62		0.37	
*Model 3*										
Distrust in clinics	1.29	(1.08,1.54)	1.90	(0.98,3.67)	2.62	(1.27,5.37)	2.27	(1.67,3.09)	1.85	(1.38,2.48)
Clinic VFM^b^	1.02	(0.83,1.26)	1.09	(0.60,2.00)	1.24	(0.73,2.11)	1.32	(1.08,1.62)	1.07	(0.80,1.44)
Clinic convenience	0.91	(0.78,1.06)	0.61	(0.37,1.00)	1.05	(0.71,1.57)	0.76	(0.63,0.92)	1.08	(0.86,1.35)
Clinic competence	0.93	(0.76,1.14)	0.63	(0.34,1.16)	0.65	(0.34,1.24)	0.76	(0.56,1.02)	0.98	(0.72,1.32)
Distrust in hospitals	0.79	(0.67,0.94)	0.52	(0.31,0.88)	0.55	(0.33,0.91)	0.52	(0.38,0.69)	0.64	(0.50,0.82)
Hospital VFM^b^	1.06	(0.90,1.25)	1.33	(0.96,1.85)	1.48	(0.92,2.40)	1.18	(0.98,1.42)	0.98	(0.80,1.20)
Hospital convenience	1.16	(1.03,1.30)	1.36	(0.99,1.87)	0.90	(0.64,1.27)	1.12	(0.94,1.32)	0.94	(0.78,1.14)
Hospital competence	1.09	(0.91,1.29)	0.99	(0.50,1.97)	0.88	(0.40,1.92)	0.89	(0.69,1.13)	1.05	(0.77,1.44)
Intercept variance	0.31		0.43		0.58		0.59		0.37	
*Model 4*										
Distrust in clinics	1.29	(1.08,1.53)	1.94	(0.99,3.78)	2.64	(1.27,5.47)	2.31	(1.70,3.15)	1.86	(1.41,2.47)
Clinic VFM^b^	1.01	(0.81,1.25)	1.03	(0.55,1.95)	1.24	(0.72,2.14)	1.31	(1.06,1.63)	1.07	(0.79,1.44)
Clinic convenience	0.90	(0.77,1.06)	0.61	(0.37,1.00)	1.08	(0.73,1.61)	0.76	(0.63,0.93)	1.09	(0.87,1.38)
Clinic competence	0.93	(0.75,1.14)	0.63	(0.34,1.17)	0.63	(0.34,1.19)	0.74	(0.55,1.00)	0.98	(0.73,1.33)
Distrust in hospitals	0.83	(0.71,0.98)	0.55	(0.32,0.95)	0.56	(0.34,0.92)	0.54	(0.39,0.74)	0.64	(0.50,0.81)
Hospital VFM^b^	1.05	(0.89,1.23)	1.30	(0.94,1.81)	1.46	(0.90,2.38)	1.16	(0.96,1.39)	0.98	(0.79,1.21)
Hospital convenience	1.16	(1.03,1.30)	1.34	(0.98,1.82)	0.87	(0.62,1.24)	1.11	(0.94,1.32)	0.94	(0.77,1.14)
Hospital competence	1.06	(0.89,1.27)	0.94	(0.48,1.81)	0.85	(0.39,1.87)	0.87	(0.68,1.17)	1.04	(0.76,1.43)
Distrust Central government/CCP	0.89	(0.74,1.07)	1.05	(0.71,1.56)	1.24	(0.81,1.90)	0.97	(0.77,1.24)	1.05	(0.80,1.36)
Distrust local government	0.92	(0.80,1.05)	0.75	(0.54,1.03)	0.87	(0.58,1.30)	0.87	(0.72,1.05)	0.92	(0.74,1.13)
Distrust media	0.99	(0.88,1.13)	0.86	(0.60,1.23)	0.73	(0.52,1.03)	0.96	(0.80,1.15)	1.18	(0.94,1.50)
Distrust most people	1.03	(0.85,1.26)	1.30	(0.83,2.02)	1.04	(0.62,1.75)	0.92	(0.73,1.16)	0.72	(0.56,0.94)
Intercept variance	0.30		0.46		0.60		0.57		0.35	

Source: authors’ survey as reported in Table 1.

^a^All models include sex, age, education, income, whether or not insured, self-assessed health and (non-agricultural or agricultural) household registration.

^b^Value-for-money.

## Results

### Greater distrust in clinics than in hospitals

We found distrust in different types of health-care providers varied substantially (Table 2). Only 6% of respondents said that they distrusted hospitals (at all levels), but over a quarter (26%) reported distrust in the providers we have grouped together and labelled ‘clinics’. In fact, distrust in hospitals (mean 1.78, SD 0.51) was not only substantially lower than distrust in clinics (2.19, SD 0.51), but also lower than distrust in local government (2.35, SD 0.80), the media (2.24, SD 0.66), and ‘people in general’ (40%), and closer to the low levels of distrust in the central government and CCP (1.64, SD 0.60) (Table 3).

Distrust in clinics and hospitals varied across the sample according to predisposing and enabling characteristics. Thus, distrust in clinics correlated negatively with age at −0.10, but positively with education at 0.17, with non-agricultural household registration at 0.15 and with household income at 0.16 (all *P* < 0.001). There was no significant correlation of distrust in clinics with sex, whether or not the respondent held any type of insurance or with self-assessed health. Distrust in hospitals showed a weak positive correlation with non-agricultural household registration at 0.05 (*P* < 0.01), and a weak negative correlation with income at −0.04 (*P* < 0.05), but it did not correlate with any of the other predisposing and enabling characteristics.

Respondents not only distrusted clinics more, they also rated them as less competent (mean 2.74, SD 0.55) than hospitals (3.36, SD 0.49) (Table 3). They did, however find them to be substantially more convenient (mean rating 3.43, SD 0.57 vs 2.46, SD 0.76). They rated clinics and hospitals very similarly in terms of their value-for-money (mean 2.84, SD 0.58 vs 2.96, SD 0.67) (Table 3).

### High rates of actual and intended hospital utilization

Respondents’ average number of hospital visits in the previous year was high at 0.55 (Table 3) and their hospital utilization for minor symptoms was also high. Across our sample, 34.4% (1267 of 3680) and 19.0% (700 of 3680 respondents) reported having a cold and headache, respectively, in the previous 2 months. Approximately 7.6% of those suffering colds (96 of 1267) and 9.4% of those suffering headaches (66 of 700) in the last 2 months had sought care at a hospital. When asked about their intended utilization (where they would seek care first) for a minor and a major illness, 13% of the sample said they would go first to a hospital for diagnosis and treatment if they had a minor illness, and 88% said they would go first to a hospital if they had a major illness (Table 3).

### Distrust in clinics associated with more hospital utilization

Distrust in clinics was associated with more hospital visits over the last year [as shown by the event rate ratio of 1.19 (confidence interval (CI) 1.01–1.40) in Model 1, Table 4]. Higher levels of distrust in clinics were also associated with substantially higher probabilities of visiting a hospital first with cold (odds ratio, OR = 1.81; CI 1.10–2.98) and headache symptoms (OR 2.42; CI 1.35–4.32). The same strong effect of distrust in clinics was apparent for intention to visit hospital for a minor illness (OR 1.90; CI 1.43–2.52) or a major illness (OR 1.53; CI 1.14–2.04).

Although the associations between distrust in clinics and hospital utilization weakened slightly (except for intention to attend hospital for a major illness) when we included clinic performance evaluations (Model 2, Table 5), the effects remained substantial, positive and statistically significant for each dependent variable except attendance at hospital for a cold. Positive evaluations of clinics’ convenience appeared to reduce intended hospital use in the event of minor illness (OR 0.78, CI 0.65–0.94).

Adding in distrust and performance evaluations for ‘hospitals’ (Model 3, Table 5) substantially increased the strength of the associations between distrust in clinics and hospital utilization across all our dependent variables. Although distrust in hospitals correlated positively with distrust in clinics (*r* = 0.32), it was associated with a ‘decrease in’ hospital utilization, while distrust in clinics was generally associated with an ‘increase’ in hospital utilization. Overall, rather than explaining distrust in clinics, adding in trust and evaluations of hospitals reinforced the message that distrust in clinics leads to higher hospital utilization for most measures (including recent actual and intended hospital utilization).

In our final model, we introduced covariates for distrust in other institutions and general distrust—to see whether wider distrust affected the associations between distrust in clinics and hospital utilization (Model 4, Table 5). Again the strong associations remained. We found that distrust in people in general reduced intended use of hospitals for a major illness (OR 0.72, CI 0.56–0.94), but institutional and general distrust measures were otherwise insignificant.

We estimate that the Chinese population’s average additional distrust in clinics over that for hospitals increases the hospitalization use rate by between 3% and 19%. This estimate is calculated taking 2.19 (the average distrust in clinics), 1.78 (the average distrust in hospitals) and the 95% CI for the odds ratio in Model 4, which is from 1.082 to 1.531. The bottom of the range for the effect of this additional distrust is calculated as exp(ln(1.082) × (2.19–1.78))=1.03, and the top of the range is calculated as exp (ln(1.531) × (2.19–1.78))=1.19.

We re-ran analyses of our models including only those people with colds or headaches who said that their symptoms did not limit their activities at all. Here, we were trying to isolate those with the most minor symptoms to see whether distrust in clinics still associated with greater (probably ‘unnecessary’) use of hospitals. We found the odds ratios for distrust in clinics to be ‘larger’ for those with non-limiting cold symptoms (see Supplementary Table S3) and similar in magnitude for hospital attendance with a headache.

When we divided our sample into those with agricultural and those with non-agricultural household registration and analysed them separately, we found that distrust in clinics was slightly lower among people with agricultural registration than those with non-agricultural registration (2.14 vs. 2.30, *P* < 0.001 with 2238 and 1255 respondents, respectively). Among those with agricultural registration the association between distrust in clinics and hospital utilization was consistently positive and significant across all dependent variables (Supplementary Table S4), which suggests that distrust in clinics is a particularly important motivation for this group to attend hospitals. Among the non-agricultural population, distrust in clinics was a positive influence on both measures of intended attendance at hospital for a minor illness and actual attendance for a headache, but insignificant for overall number of visits, intended attendance for a major illness or actual attendance for a cold (Supplementary Table S5).

## Discussion

Our study provides novel data on distrust and utilization in China 3 years after its 2009 health-care reform programme began. The data are based on a small but nationally representative sample of the Chinese population. This enables us to explore the associations between distrust and utilization in ways that studies of only patient populations cannot. Because different measures of health-care utilization are known to have different strengths and weaknesses, we examined whether the results were consistent across a range of measures of actual and intended utilization.

Our study contributes in four important ways to understanding trust and its relationship with health-care utilization. First, while it reveals significant distrust in health-care providers across the Chinese population, it shows that there has not been the collapse in public trust some researchers have expected (Hsiao and Hu 2011). While it demonstrates distrust in clinics and other primary care facilities to be high, and it shows distrust in hospitals to be much lower.

Second, our study shows distrust to have a strong association with choice of health-care provider in a health-care system beyond the USA, the site of previous research on trust and utilization. Previous research on China—like other developing countries—has focussed on the important effects of cost, ability to pay and distance to health service provider (Wang *et al*. 2005; Liu *et al*. 2007), but has taken little account of distrust (Wagstaff *et al*. 2009). Our study indicates that distrust is likely to have a significant independent effect, with distrust in clinics increasing hospital use even when we control for insurance, ability to pay and convenience.

Our study has important policy implications. It shows high levels of distrust in clinics to be strongly associated with greater use of hospitals, including actual use of hospital care for minor symptoms (cold, headaches) and intended use in relation to minor illness. It thus suggests that policy makers can address China’s highly inefficient patterns of utilization by reducing distrust in clinics.

Our study suggests, however, that to reduce distrust policy makers need to do more than simply improve the training of medical professionals working in clinics. Previous studies indicate, but on limited evidence, that patient distrust in clinics is primarily due to perceptions of poor competence. An important contribution of our article is to show that public distrust in clinics is not explained simply by negative evaluations of their competence.

To support the current Chinese government policy of increasing clinic utilization, more research is needed to understand public distrust in these facilities and across the health-care system. In the absence of panel data on distrust and utilization, our study contributes to understanding the relationships between public distrust and the utilization of different health-care providers. It also provides a baseline for further national studies of distrust. But we cannot, on the basis of our cross-sectional data, prove that the direction of causality runs simply from distrust to utilization. We think, however, that it is plausible to interpret the associations in our data as showing distrust to affect utilization rather than the reverse: not only do we find the same strong association across all our measures of utilization, we also find strong associations between distrust in clinics and actual utilization of hospitals and think it unlikely that people’s experience of hospitals in the last year would increase their distrust in clinics. We do accept, however, that previous experiences of using clinics might have affected trust in them and thereby led indirectly to greater use of hospitals. While trust remained significant even after we allowed for evaluations which ask explicitly about historical experience, further research is needed to probe the relationships between them.

Further research might develop our measure of distrust for the Chinese health-care context. Since trust and distrust in health-care in China (as in other developing countries) has not previously been empirically researched in any depth, we preferred not to prejudge its dimensions. Our holistic measure of trust provided a unifying concept with which to compare attitudes towards a range of different types of institutions. And rather than focussing on the development of a complex scale for particular health-care contexts, we sought instead to simply assess overall levels of trust in different kinds of provider using a straightforward direct question. But having established that in China there is differential trust in health-care institutions and it has major implications for the health system, there is a strong case for developing more sophisticated scales or other measures of trust’s dimensions.

Our study is relevant beyond the boundaries of middle-income China. As we discuss above, while the sources of distrust in providers in China are not well understood, they are thought to be linked to low investment and the reliance on fee-for-service financing (Liu 2004; Blumenthal and Hsiao 2005). At the same time, weak hospital gate-keeping and the prevalence of out-of-pocket payments contribute to over-utilization of hospitals (Yip *et al*. 2012). These features of China’s health-care system are found elsewhere, particularly in low- and middle-income countries, and may spread as China increases its international cooperation in public health and health services (Lagarde and Palmer 2008; Huang 2011). For both these reasons, our study illustrates the importance of examining the relationship between distrust and health-care utilization in other parts of the developing world.

## Supplementary data

Supplementary data are available at *HEAPOL* online

Supplementary Data
