# Supplementary material for: Does distrust in providers affect health-care utilization in China?
Source: Health Policy Plan. 2016 Apr 26;31(8):1001–9. doi: 10.1093/heapol/czw024 (PMC5013779; doi:10.1093/heapol/czw024)
Supplement: Supplementary Data [file supp_31_8_1001__index.html]

Does distrust in providers affect health-care utilization in China? — Does distrust in providers affect health-care utilization in China? — Supplementary Data 

# Does distrust in providers affect health-care utilization in China?

## Supplementary Data

files

- Supplementary Data - docx file
- Supplementary Data - docx file
