# Supplementary material for: Does distrust in providers affect health-care utilization in China?
Source: Health Policy Plan. 2016 Apr 26;31(8):1001–9. doi: 10.1093/heapol/czw024 (PMC5013779; doi:10.1093/heapol/czw024)
Supplement: Supplementary Data [file supp_czw024_Supplementary_Material.docx]

**Supplementary Material**

*Appendix 1: The Survey*

Stratification took place in stages. We first divided the country into three official macro-regions: Eastern, Central and Western. We then divided each macro-region into urban and rural administrative areas, designating municipal districts and county-level cities as “urban,” and counties as “rural.” (We coded separately for our respondents’ household registration as “agricultural” or “non-agricultural,” something that continues to affect health-care insurance and other social entitlements even after the actual place of residence has changed.) We then selected at random sixty primary sampling units (PSUs) corresponding to county-level administrative divisions across the six regional/administrative segments with probability proportionate to population. Within each PSU, three half-square minutes (HSMs) of latitude and longitude were chosen with probability proportionate to population density, and within each of these, again proportionate to population density, a number of spatial square seconds (SSS) corresponding to 90m x 90m squares was selected at random. Within each SSS, all dwellings were enumerated, and 27 were selected in each HSM by systematic sampling. One important advantage of this method is that it enabled us to include migrants in our sample – unlike the many surveys in China that rely on local government registers of residents (Landry & Shen 2005).

Interviewers visited each selected dwelling to conduct face-to-face interviews. Within each dwelling they identified respondents using the Kish method (Kish 1949). Field supervisors collected, checked and verified completed questionnaires on location during database creation using RCCC’s standard quality control practices. They checked that the interviewer had entered the correct address, used the Kish grid to select interviewees, interviewed the selected interviewee, and obtained answers to all questions. They also checked that interviewee responses were clear and logical. The supervisors for the project were all employees of, and trained by, the RCCC at Peking University. The interviewers for the project were college students in the surveyed area and were trained according to the RCCC Interviewer Manual by their supervisors. Supervisor and interviewer training covered the project’s background, interview techniques, the specific requirements of the project, the use of GPS in the sample area selection process, procedures for selecting addresses and interviewees, review of each question in the questionnaire, as well as procedures for project implementation, quality control, codes of conduct, and safety protocols.

Specialists at RCCC created the database using specialized data entry software and the double data entry method. Three Peking University graduate students were responsible for verifying the validity of both complete and incomplete questionnaires. They checked whether the interviewees were properly selected and whether the interviews were indeed conducted on the selected individuals. Experienced research assistants at RCCC led data cleaning, verified that data entry procedures were correctly followed, searched for incorrect data and logical fallacies within the responses, and after these were corrected, separate individuals checked and double checked for errors and logical fallacies.

*Appendix 2: Questionnaire design, pilot and quality control*

To develop the questionnaire, we analyzed discussions from nine focus groups in urban and rural China to better understand ordinary citizens’ views and experiences of the health-care system and improve research design. These focus groups were conducted on our behalf by native speakers from RCCC, and two members of our team (and two authors of this paper) had the linguistic skills to read the transcripts in both English and Chinese. We also reviewed existing survey instruments against our research questions. This enabled us to identify possible items for inclusion or adaptation, as well as to determine where we needed to develop new questions. We drew where possible on instruments from major China health surveys including the China Health and Retirement Longitudinal Study (CHARLS), the China Economic, Population, Health and Nutrition Survey (CHNS), and the China Urban Labor Survey (CULS). Native Chinese speakers with expertise in the Chinese health system and health policy translated our draft questionnaire into modern standard Chinese, and we then back-translated it into English. We used the existing Chinese wording where an instrument had already been used in that language in a major previous survey. We discussed individual questions at length with native speakers with the aim of ensuring that the translation both captured our concepts and intentions and was likely to make sense to interviewees in China. On this basis, we prepared a draft questionnaire. We then piloted the survey near Beijing using face-to-face interviews with a sample of 50 respondents, 26 with “non-agricultural” and 24 with “agricultural” household registration. We analyzed responses to the pilot and feedback from our interviewers on interviewees’ level of understanding and any questions that caused interviewee confusion or difficulty. We then used this information to revise the questionnaire so as to further ensure clarity and comprehensibility.

In our full survey, interviewers assessed respondents’ cooperativeness, level of understanding, and level of interest in the interview using a five point scale. Scores at or above the mid-point on these measures were 97.7% (or 3,595 of 3,680 respondents), 95.4% (3,512 respondents) and 92.1% (3,389) respectively. Interviewers also assessed respondents’ level of doubt about the interview before it started and recorded their impression of the reliability of respondents’ replies on a three point scale. Scores at or above the mid-point were 97.3% (3,583 respondents) and 96.1% (3,538) respectively.

*Appendix 3: Missing Data Handling*

## To deal with missing data, we conducted our analyses using both the “complete case” (listwise deletion) method and by pooling estimates obtained from multiple imputed datasets. Using complete case analysis reduces the sample size substantially, and so increases standard errors and may result in non-random restrictions of the sample (Allison 2002). We therefore also used multiple imputation in SPSS to create six complete datasets using fully conditional specification (FCS), an iterative Markov Chain Monte Carlo (MCMC) method that is appropriate when the pattern of missing data is arbitrary (Van Buuren et al 2006). We imputed values to all variables having at least one per cent of cases missing, using all variables having at least ten percent of cases not missing, producing five complete data sets with 3,680 cases each. The complete list of over 160 variables used in the imputation is available from the authors. To build our model, we first conducted exploratory analysis with one imputation of missing data. Then, when we had constructed our models, we re-ran it using the five remaining imputations to obtain pooled parameter estimates. Because the pooling process takes into account variation amongst five imputations, it tends to increase standard errors and lower test statistics compared to those obtained from a single imputation, and therefore offers a more stringent test of the significance of individual parameters than could be obtained without pooling (Allison 2002). We compared the results of our regressions using both multiple imputation and listwise deletion methods, and found them to be substantively the same.
