# Supplementary material for: Does distrust in providers affect health-care utilization in China?
Source: Health Policy Plan. 2016 Apr 26;31(8):1001–9. doi: 10.1093/heapol/czw024 (PMC5013779; doi:10.1093/heapol/czw024)
Supplement: Supplementary Data [file supp_czw024_Supplementary_Tables.docx]

**Supplementary tables**

| Supplementary Table 1. Principal Components Analysis of Trust Variable**s** | | |
| --- | --- | --- |
|  | **Component loadings^a^** | |
|  | 1 | 2 |
| Trust village health clinic | **∙87** | ∙03 |
| Trust small clinic | **∙84** | ∙01 |
| Trust community health service station | **∙82** | ∙19 |
| Trust township, town or street health centre | **∙74** | ∙30 |
| Trust pharmacy | ∙44 | ∙27 |
| Trust prefecture level hospital | ∙13 | **∙93** |
| Trust province level hospital | ∙07 | **∙89** |
| Trust county/city/district hospital | ∙26 | **∙81** |
| Eigen value | 3∙67 | 1∙79 |
| % variance explained | 45∙88 | 22∙35 |
| Source: authors’ survey as reported in Table 1.  ^a^ Components rotated by Varimax method with Kaiser normalization; highest loadings for each variable are shown in bold. | | |

| Supplementary Table 2. Principal Components Analysis of Performance Evaluations | | | | | | |
| --- | --- | --- | --- | --- | --- | --- |
|  | **Component loadings^a^** | | | | | |
| Skills and experience: village clinics | **0∙87** | 0∙22 | 0∙07 | 0∙02 | 0∙03 | -0∙01 |
| Skills and experience: small clinics | **0∙83** | 0∙21 | 0∙05 | 0∙03 | 0∙06 | -0∙03 |
| Skills and experience: community health service stations | **0∙82** | 0∙21 | 0∙00 | 0∙05 | 0∙07 | 0∙15 |
| Skills and experience: township, town or street health centre | **0∙71** | 0∙22 | 0∙00 | 0∙06 | 0∙05 | 0∙29 |
| Value for money: village clinics | 0∙26 | **0∙85** | 0∙10 | 0∙02 | -0∙01 | 0∙04 |
| Value for money: community health service stations | 0∙22 | **0∙84** | 0∙07 | 0∙14 | 0∙04 | 0∙08 |
| Value for money: small clinics | 0∙26 | **0∙82** | 0∙10 | 0∙00 | 0∙02 | 0∙02 |
| Value for money: township, town or street health centre | 0∙14 | **0∙72** | 0∙05 | 0∙34 | 0∙05 | 0∙09 |
| Convenience: village clinics | 0∙09 | 0∙08 | **0∙84** | 0∙00 | -0∙13 | 0∙07 |
| Convenience: community health service stations | -0∙01 | 0∙07 | **0∙82** | 0∙06 | 0∙12 | 0∙13 |
| Convenience: small clinics | 0∙07 | 0∙08 | **0∙80** | 0∙00 | -0∙11 | 0∙10 |
| Convenience: township, town or street health centre | -0∙03 | 0∙07 | **0∙68** | 0∙04 | 0∙36 | 0∙06 |
| Value for money: prefecture level hospitals | 0∙02 | 0∙11 | 0∙03 | **0∙92** | 0∙09 | 0∙12 |
| Value for money: province level hospitals | 0∙04 | 0∙03 | 0∙03 | **0∙91** | 0∙07 | 0∙07 |
| Value for money: county/city/district hospitals | 0∙06 | 0∙22 | 0∙03 | **0∙81** | 0∙10 | 0∙17 |
| Convenience: prefecture level hospitals | 0∙07 | 0∙04 | -0∙04 | 0∙08 | **0∙89** | 0∙00 |
| Convenience: province level hospitals | 0∙11 | 0∙02 | -0∙09 | 0∙08 | **0∙82** | -0∙01 |
| Convenience: county/city/district hospitals | 0∙00 | 0∙01 | 0∙22 | 0∙07 | **0∙77** | 0∙02 |
| Skills and experience: prefecture level hospitals | 0∙09 | 0∙07 | 0∙13 | 0∙12 | 0∙00 | **0∙89** |
| Skills and experience: province level hospitals | -0∙04 | 0∙02 | 0∙15 | 0∙10 | -0∙01 | **0∙85** |
| Skills and experience: county/city/district hospitals | 0∙28 | 0∙10 | 0∙07 | 0∙14 | 0∙04 | **0∙73** |
| Eigen values | 5∙43 | 2∙68 | 2∙42 | 2∙10 | 1∙73 | 1∙18 |
| % variance explained | 25∙84 | 12∙77 | 11∙53 | 10∙00 | 8∙24 | 5∙61 |
| Cronbach’s alpha for highest loading items | ∙90 | ∙91 | ∙85 | ∙93 | ∙85 | ∙85 |
| Source: as reported in Table 1. ^a^ Rotated by Varimax method with Kaiser normalization; highest loadings shown in bold. | | | | | | |

| Supplementary Table 3. Multivariate Analysis: Minor condition Requiring No Change in Activities | | | | | | | | | | | |
| --- | --- | --- | --- | --- | --- | --- | --- | --- | --- | --- | --- |
|  | **Went to hospital for cold** | | **Went to hospital for headache** | | | | | | | | |
|  | Event rate ratios with 95% confidence interval (lower, upper) | | | | | | | | | | |
| Male | 0.83 | (0.53,1.31) | | 1.17 | | (0.68,2.02) | | | | | |
| Insured | 1.66 | (0.57,4.82) | | 1.25 | | (0.40,3.92) | | | | | |
| Non-agricultural h/h reg | 1.31 | (0.77,2.23) | | 0.64 | | (0.33,1.22) | | | | | |
| Age in deciles | 1.05 | (0.89,1.25) | | 0.91 | | (0.72,1.15) | | | | | |
| Self-assessed health | 0.66 | (0.49,0.89) | | 0.74 | | (0.54,1.00) | | | | | |
| Education | 1.04 | (0.72,1.50) | | 1.10 | | (0.80,1.52) | | | | | |
| Household income | 1.01 | (1.00,1.02) | | 1.00 | | (0.99,1.02) | | | | | |
| Distrust in clinics | 2.10 | (1.04,4.24) | | 2.36 | | (1.15,4.87) | | | | | |
| Intercept variance: |  |  | |  |  | |  |  |  |  |  |
| for null model | 0.61 |  | |  | 0.95 | |  |  |  |  |  |
| for fitted model | 0.54 |  | |  | 0.87 | |  |  |  |  |  |
| Source: authors’ survey as reported in Table 1. | | | | | | | | | | | |

| Supplementary Table 4. Multivariate Analysis: Agricultural Household Registration Only | | | | | | | | | | |
| --- | --- | --- | --- | --- | --- | --- | --- | --- | --- | --- |
|  | **N hospital visits** | | **Went to hospital for cold** | | **Went to hospital for headache** | | **Would go to hospital for minor illness** | | **Would go to hospital for major illness** | |
|  | Event rate ratios with 95% confidence interval (lower, upper) | | | | | | | | | |
| Male | 1.03 | (0.76,1.39) | 1.24 | (0.67,2.29) | 1.51 | (0.91,2.50) | 1.03 | (0.76,1.39) | 0.80 | (0.68,0.95) |
| Insured | 1.71 | (0.93,3.14) | 4.16 | (0.88,19.78) | 2.67 | (0.62,11.56) | 1.71 | (0.93,3.14) | 1.11 | (0.61,2.04) |
| Age in deciles | 0.92 | (0.82,1.03) | 1.02 | (0.81,1.29) | 0.97 | (0.75,1.26) | 0.92 | (0.82,1.03) | 0.95 | (0.86,1.05) |
| Self-assessed health | 0.92 | (0.77,1.10) | 0.63 | (0.44,0.90) | 0.73 | (0.53,1.01) | 0.92 | (0.77,1.10) | 0.97 | (0.86,1.11) |
| Education | 1.19 | (1.01,1.40) | 1.06 | (0.73,1.53) | 1.34 | (0.98,1.85) | 1.19 | (1.01,1.40) | 1.06 | (0.86,1.30) |
| Household income | 1.00 | (1.00,1.01) | 1.01 | (1.00,1.02) | 1.00 | (0.98,1.02) | 1.00 | (1.00,1.01) | 1.00 | (1.00,1.01) |
| Distrust in clinics | 2.38 | (1.60,3.56) | 2.88 | (1.15,7.21) | 2.31 | (1.09,4.86) | 2.38 | (1.60,3.56) | 1.59 | (1.10,2.28) |
| Intercept variance: |  |  |  |  |  |  |  |  |  |  |
| for null model | 0.47 |  | 0.60 |  | 0.93 |  | 1.22 |  | 0.48 |  |
| for fitted model | 0.42 |  | 0.54 |  | 0.78 |  | 0.96 |  | 0.49 |  |
| Source: authors’ survey as reported in Table 1. | | | | | | | | | | |

| Supplementary Table 5. Multivariate Analysis: Non-agricultural Household Registration Only | | | | | | | | | | | | | |
| --- | --- | --- | --- | --- | --- | --- | --- | --- | --- | --- | --- | --- | --- |
|  | **N hospital visits** | | **Went to hospital for cold** | | | **Went to hospital for headache** | | | **Would go to hospital for minor illness** | | | **Would go to hospital for major illness** | |
|  | Event rate ratios with 95% confidence interval (lower, upper) | | | | | | | | | | | | |
| Male | 0.97 | (0.79,1.20) | | 0.91 | (0.56,1.45) | | 0.75 | (0.34,1.67) | | 0.86 | (0.67,1.11) | 1.25 | (0.82,1.93) |
| Insured | 1.50 | (0.89,2.53) | | 1.32 | (0.43,4.06) | | 0.94 | (0.31,2.90) | | 1.56 | (0.97,2.50) | 2.39 | (1.31,4.35) |
| Age in deciles | 1.15 | (1.04,1.26) | | 1.05 | (0.82,1.35) | | 1.08 | (0.82,1.43) | | 1.08 | (0.97,1.21) | 0.93 | (0.79,1.08) |
| Self-assessed health | 0.60 | (0.49,0.75) | | 0.92 | (0.64,1.33) | | 0.96 | (0.59,1.55) | | 1.09 | (0.91,1.31) | 0.93 | (0.73,1.19) |
| Education | 1.10 | (0.98,1.25) | | 1.18 | (0.74,1.88) | | 0.88 | (0.60,1.30) | | 1.17 | (0.92,1.50) | 1.17 | (0.88,1.57) |
| Household income | 1.00 | (1.00,1.00) | | 1.00 | (1.00,1.02) | | 1.01 | (1.00,1.02) | | 1.00 | (1.00,1.01) | 1.00 | (1.00,1.01) |
| Distrust in clinics | 1.16 | (0.94,1.44) | | 1.43 | (0.81,2.52) | | 2.53 | (1.18,5.42) | | 1.57 | (1.06,2.30) | 1.31 | (0.83,2.04) |
| Intercept variance: |  |  | |  |  | |  |  | |  |  |  |  |
| for null model | 0.41 |  | | 0.31 |  | | 0.59 |  | | 0.47 |  | 0.41 |  |
| for fitted model | 0.40 |  | | 0.22 |  | | 0.61 |  | | 0.47 |  | 0.34 |  |
| Source: authors’ survey as reported in Table 1. | | | | | | | | | | | | | |
